# Down-Regulated Exosomal MicroRNA-221 – 3p Derived From Senescent Mesenchymal Stem Cells Impairs Heart Repair

**DOI:** 10.3389/fcell.2020.00263

**Published:** 2020-05-05

**Authors:** Ling Sun, Wenwu Zhu, Pengcheng Zhao, Jian Zhang, Yao Lu, Yeqian Zhu, Wei Zhao, Yaowu Liu, Qiushi Chen, Fengxiang Zhang

**Affiliations:** ^1^Section of Pacing and Electrophysiology, Division of Cardiology, The First Affiliated Hospital with Nanjing Medical University, Nanjing, China; ^2^Department of Cardiology, The Affiliated Changzhou No. 2 People’s Hospital of Nanjing Medical University, Changzhou, China; ^3^Department of Cardiology, Zhongda Hospital of Southeast University, Nanjing, China

**Keywords:** microRNA-221-3p, exosomes, mesenchymal stem cells, senescence, acute myocardial infarction

## Abstract

The composition and biological activity of donor cells is largely determined by the exosomes they secrete. In this study, we isolated exosomes from young (Young-Exo) and aged (Age-Exo) mesenchymal stem cells (MSCs) and compared their regeneration activity. Young Exo MSCs were more efficient than Aged-Exo at promoting the formation of endothelial tube, reducing fibrosis, and inhibiting apoptosis of cardiomyocytes *in vitro*; and improving cardiac structure and function *in vivo* in the hearts of rats following myocardial infarction (MI). MicroRNA sequencing and polymerase chain reaction (PCR) analysis revealed that miR-221-3p was significantly down-regulated in Aged-Exo. The aged MSCs were rejuvenated and their reparative cardiac ability restored when miR-221-3p was overexpressed in Aged-Exo. The protective effect was lost when miR-221-3p expression was knocked down in Young-Exo. These effects of miR-221-3p were achieved through enhancing Akt kinase activity by inhibiting phosphatase and tensin homolog (PTEN). In conclusion, exosomal miR-221-3p secreted from Aged MSCs attenuated the function of angiogenesis and promoted survival of cardiomyocytes. Up-regulation of miR-221-3p in aged MSCs improved their ability of angiogenesis, migration and proliferation, and suppressed apoptosis via the PTEN/Akt pathway.

## Introduction

Mesenchymal stem cell (MSCs) transplantation has enabled great progress in the treatment of ischemic heart diseases (Kanelidis et al., 2017). Nonetheless their cardioprotective effect declines with cell aging and this limits the application of autologous cell therapy in elderly patients with ischemic heart disease (Fisher et al., 2015; Nguyen et al., 2016). In addition, *in vitro* amplification of MSCs is necessary prior to their clinical application and inevitably leads to replicative aging process (Mathiasen et al., 2015; Guijarro et al., 2016). Accumulating evidence shows that aging affects the functions of MSCs, including differentiation, proliferation and migration, as well as angiogenic potential, and in turn reduces their clinical efficacy (Malaise et al., 2019; Zhang et al., 2019; Wang et al., 2020). There is an urgent need to explore strategies that will enable functional recovery of elderly MSCs.

Recent studies have shown that MSCs protect the heart mainly through secretion of paracrine factors such as exosomes (Exos) (Boulanger et al., 2017; Heallen and Martin, 2018; Park et al., 2019). Exosomes originate intracellularly from a variety of cell types and transfer bioactive molecules such as miRNAs and proteins between cells (Davis, 2016). When donor cells are stimulated by the environment, the content of the exosomes will change and so also their biological effect (Boriachek et al., 2018).

miR-221-3p is a well-known miRNA that promotes cell survival and proliferation during tumorigenesis (Zhang et al., 2010; Yuan et al., 2013; Fornari et al., 2017). Circulating miR-221 has also been shown to be increased in patients with acute myocardial infarction (MI) and hypertrophic cardiomyopathy (Huang et al., 2020). Up-regulation of miR-221-3p inhibits autophagy in cardiomyocytes (Chen et al., 2016) while down-regulation enables profibrotic signaling in patients with dilated cardiomyopathy (Verjans et al., 2018). These data suggest that miR-221-3p plays a vital role in cardiovascular diseases. We performed pretest of miRNA sequencing and established that exosomal miR-221-3p was much higher in young MSCs and aged MSCs. Therefore, in this study we aimed to compare the cardiac repair effects of exosomal miR-221-3p secreted from young and aged MSCs, and to explore the possible underlying mechanism.

## Materials and Methods

### Cells Culture

Human bone marrow was harvested from the posterior superior iliac spine of aged donors [70–80 years old, male (*n* = 3) and female (*n* = 3)] or young donors [20–25 years old, male (*n* = 3) and female (*n* = 3)]. All MSCs used for experiments in this study were between passages 4 and 6. Human bone marrow derived-MSCs were cultured in α-minimal essential medium (α-MEM) with 10% fetal bovine serum (FBS). An H9c2 cardiomyoblast cell line (ATCC) and human umbilical vein endothelial cells (HUVECs) were cultured in DMEM containing 10% FBS. All media and reagents for cell culture were purchased from Gibco (Carlsbad, United States). For normal culture, cells were incubated at 37°C, 21% O_2_, and 5% CO_2_. For hypoxia and serum deprivation (H/SD) condition, cells were cultured at 1% O_2_, 5% CO_2_, 94% N_2_ and serum deprivation condition.

### Exosome Extraction and Characterization

The supernatants of cells were collected and Exos extracted using Exosome Isolation Reagent (Ribobio, Guangzhou, China). Transmission electron microscopy (TEM) was used to observe the morphology of Exos. Briefly, Exos were fixed with 1% glutaraldehyde, then coated on a copper mesh and stained with 1% phosphotungstic acid. Samples were observed with a JEM-2100 transmission electron microscope (JEOL, Tokyo, Japan). Nanoparticle tracking analysis (NTA) was used to evaluate the size and distribution of Exos. We recorded and tracked the Brownian motion of Exos in PBS (Invitrogen, Carlsbad, CA, United States). The Stoke-Einstein equation was used to obtain the size distribution data. A ZetaView PMX 110 system (Particle Metrix, Germany) was used for NTA.

### Western Blotting

Western blotting was performed using a gel documentation system (iBrightCL1000, Invitrogen and Image Lab Software version 3.0), and a standard protocol as previously described (Zhang et al., 2019). The primary antibodies were anti-cyclin dependent kinase inhibitor 2A (P16) (80772, Cell Signaling Technology, United States), anti-cyclin dependent kinase inhibitor 1A (P21) (2947, Cell Signaling Technology), P53 (21083, Signalway Antibody, United States), PTEN (ab31392, abcam, United Kingdom), anti-phosphorylated-AKT (p-AKT) (4060, Cell Signaling Technology) and anti-AKT (4691, Cell Signaling Technology); Cleaved caspase-3 (29034, Signalway Antibody), Bcl-2 (ab196495, abcam), vascular endothelial growth factor (VEGF, ab52917, abcam), anti-glyceraldehyde-3-phosphate dehydrogenase (GAPDH) (5174, Cell Signaling Technology); TSG101 (14497, Proteintech, United States), CD63 (25682, Proteintech), CD81 (66866, Proteintech) and horseradish peroxidase-conjugated were secondary antibodies (Biosharp, China).

### SA-β-Gal Staining Assay

Cell senescence was determined using a Senescence-β-Galactosidase Cell Staining Kit (Cell Signaling Technology) according to the manufacturer’s instructions. Briefly, we rinsed the plate with PBS and fixed cells in staining fixatives for 15 min at room temperature. Fixed cells were then washed twice with PBS and stained with fresh SA-β-gal staining solution overnight at 37°C. Cells that stained positive for SA-β-Gal were quantified and analyzed.

### Cell Cycle Assay

Flow cytometry (Becton Dickinson, United States) was used for cell cycle assay. Briefly, 1 × 10^6^ cells were collected and washed twice with PBS. After staining with 1 ml DNA staining solution and 10 μL permeabilization solution of Cell Cycle Staining Kit (KeyGen Biotech, Nanjing, China) for 30 min, cells were evaluated and further analyzed using FlowJo software version 10.0 (Tree Star, United States).

### Cell Viability Analysis

CCK-8 Kit (Dojindo Laboratories, Kumamoto, Japan) was used for cell viability assays. Briefly, cells were seeded at a density of 5 × 10^3^ cells/well in a 96 well plate and cultured for 24 h after which 10 μL of CCK-8 solution was added to each well. After incubation for a further 2 h, the absorbance of each well (450 nm) was determined by a microplate reader (Synergy, United States).

### Identification of Surface Antigen Using Flow Cytometry

Both Aged and Young MSCs were characterized by their cell surface antigen expression and trilineage differentiation capability using flow cytometry. Antibodies used for MSC characterization were anti-CD105-APC (17-1057-41; Thermo Fisher Scientific), anti-CD44-APC (17-0441-81; Thermo Fisher Scientific), anti-CD73-APC (17-0739-41; Thermo Fisher Scientific), anti-CD90-APC (17-0909-41; Thermo Fisher Scientific), anti-CD45-APC (17-9459-41; Thermo Fisher Scientific), and anti-CD31-APC (17-0319-41; Thermo Fisher Scientific).

### Exosome Uptake by H9c2 Cells and HUVECs

To evaluate MSC-Exo uptake by H9c2 cells and HUVECs, Dil (red fluorescent dye, C1036, Beyotime, China) was used to label Exos. Then Exos and recipient cells were co-cultured for 6 and 24 h at 37°C, after which they were washed with PBS and fixed with 4% paraformaldehyde for 20 min. Nuclei were then stained with 6-diamidino-2-phenylindole (DAPI) (0.5 g/ml; Beyotime) for 10 min and observed under a confocal microscope.

### Cell Apoptosis and Proliferation and Assay

Flow cytometry was used to assess cell apoptosis. H9c2 cells or HUVECs were cultured overnight at a seeding density of 1 × 10^5^/6-well tissue culture plates and treated with Exos or PBS before H9c2 were subjected to hypoxia. To quantify the apoptotic cells, cells were washed with PBS and stained using an annexin V-FITC and propidium iodide (PI) apoptosis kit (KeyGen Biotech, China). The apoptotic cells were analyzed by Flowjo Software version 10.0 (Tree Star, United States).

Cell proliferation was assessed by Edu assay using a kFluor488-EDU Kit (KeyGen Biotech). Briefly, 1 × 10^5^ cells were seeded in well plates and 1:1000 dilution of Edu-labeling reagent added. Cells were then fixed for 48 h and incubated with 0.1 ml 3% bovine serum albumin (BSA) in PBS. Then 0.5% Triton X-100 was added for 20 min and then Click-iT Edu reagent. The nuclei were stained with DAPI. Fluorescence microscopy was used to observe the Edu positive cells and Image J software (National Institutes of Health, NIH) was used for further analysis.

### Migration Assay

HUVECs were cultured in a 6-well plate, and a fusion layer scratched using a P200 pipette tip. Cells were then washed and incubated after addition of 100 μg/well Exos. Images were taken before and 24 h after incubation and Image J software (NIH) used to determine any reduction in scratched area size.

### Tube Formation Assay

Tube formation assay was performed to assess the angiogenic ability of HUVECs. Briefly, HUVECs were treated with PBS or exosomes extracted from young (Young-Exo) and aged MSCs (Aged-Exo) for 24 h. Then cells were washed with PBS and seeded (30,000 cells/well) in 96 well plates coated with growth-factor reduced Matrigel (Corning, United States). After 6 h, capillary-like tube formation was observed and photographed. Tube length and number of branches were analyzed with Image J software (NIH).

### MI Model, Histological Analysis, and Immunofluorescence Staining

Sprague-Dawley (SD) rats (Male, 200–220 g weight) were anesthetized and ventilated via an orotracheal tube and rodent ventilator. Left anterior descending artery (LAD) was ligated and Exos (50 μL, 1 μg/μL) or PBS injected around the infarcted region. Echocardiography (Vevo 3100) was performed 2 and 4 weeks later to determine left ventricular ejection fraction (LVEF) and left ventricular fractional shortening (LVFS) (Zhang et al., 2019).

Rats were sacrificed 4 weeks post MI. Hematoxylin-Eosin (HE) stain was used to evaluate inflammatory cell infiltration. The fibrotic and collagen area post MI was evaluated by Masson’s Trichrome stain and Sirius Red stain.

CD31 immunofluorescence staining was performed as previously described (Zhang et al., 2019). Terminal deoxynucleotidyl transferase nick-end labeling (TUNEL) staining (Roche, United States) was used to assess cell apoptosis. The primary antibodies were anti-CD31 (ab7388; Abcam, United Kingdom), and anti–α-sarcomeric actin (α-actin, a7811, Sigma-Aldrich). DAPI was used for nuclear counter staining. Images were taken using a fluorescent microscope (Zeiss, German) and Image J software (NIH) used for further analysis.

### Distribution of Dil-Labeled Exosomes in Infarcted Hearts of Rats

A 50 μL (1 μg/μL) solution of Dil-labeled Exos was injected into the injured rat hearts. Rats were sacrificed 6 h after injection and the heart removed. Heart tissue was dehydrated, frozen, and sliced into 6 μm slices. Antibodies used for immunofluorescence staining were α-actin (Sigma-Aldrich), and anti-CD31 (Abcam, United Kingdom). The nuclei were stained with DAPI and cells observed under fluorescence microscopy (Zeiss, Germany).

### Exosomal MicroRNA Sequencing and Real-Time RT-PCR Validation

Exosome microRNAs were sequenced in Young-Exo and Aged-Exo. Differentially expressed microRNAs were identified by log_2_ |(Fold Change)| > 1 and *P* < 0.05 with the threshold set for up and down regulated microRNAs.

Total RNA was isolated by TRIzol (Life technologies, United States). RNA concentration was quantitated by the Nano Drop ND-2000 Spectrophotometer (Nano Drop Technologies, Wilmington, DE, United States) and cDNAs were synthesized using PrimeScript^TM^ RT reagent kit (TaKaRa, Japan). Real-time polymerase chain reaction (PCR) was performed by SYBR Premix Ex Taq qRT–PCR assays (TaKaRa, Japan) with microRNAs, Cel-mir-39, and U6 specific primers (Genscript, Nanjing, China) under 7900HT Real-Time PCR Detection System (Thermo Fisher Scientific, United States). Cel-mir-39 (exosomal) and U6 (cellular) served as external or internal standard to normalize the miRNA expression level using 2^–ΔΔ^^Ct^ method. Primer sequences were listed in Supplementary Table S1.

### Lentiviral Package and Cell Transfection

Lentiviral plasmids encoding miR-221 and its negative control were obtained from GENECHEM (Shanghai, China). Lentivirus (hU6 – MCS – Ubiquitin – EGFP – IRES – puromycin) transfected MSCs had a multiplicity of infection (MOI) of 60 for miR-221 and 80 for its negative control. Cells were selected with purinomycin (0.75 μg/ml) for 3 days. miR-221-3p mimics, inhibitor and their negative control were obtained from Ribobio (Guangzhou, China). Lipofectamine2000 (Invitrogen, United States) was used for cell transfection.

### Statistical Analysis

Continuous variables and categorical variables are described as mean ± SD and percentages, respectively. Independent-Sample *T*-test was used to compare continuous variables between the two groups. One way Analysis of variance (ANOVA) followed by Tukey’s correction was used for comparison of three or more groups. All statistical tests were performed using GraphPad Prism software version 8.0, and *p* < 0.05 was considered statistically significant.

## Results

### Characterization of MSCs and Exosomes

**FIGURE 1:**
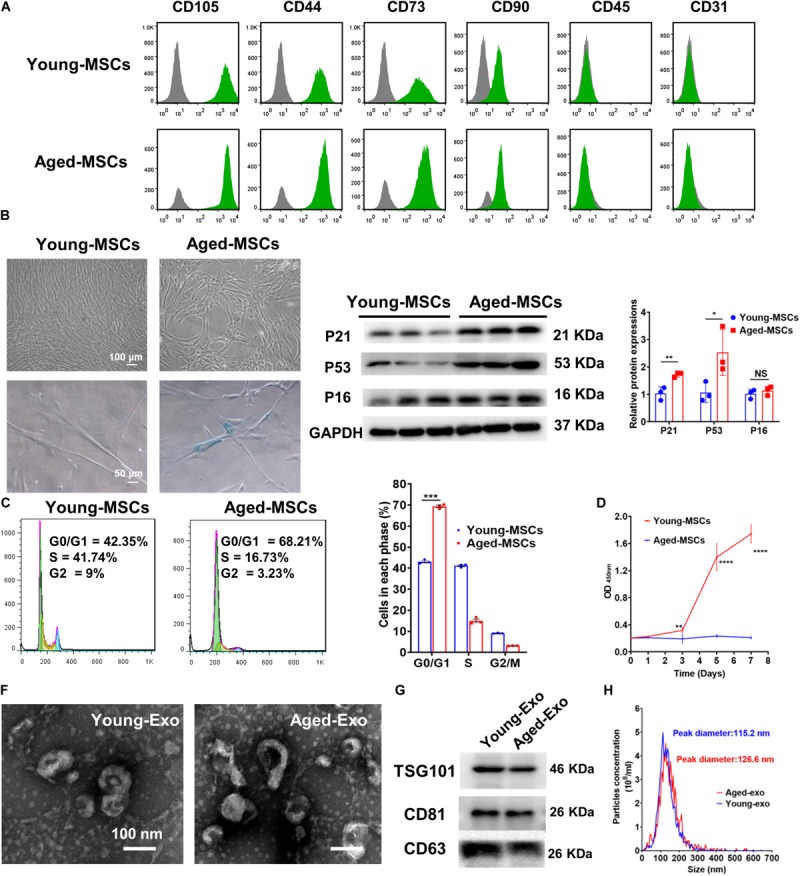
Characterization of young and aged MSCs and exosomes. **(A)** Surface marker profiling of young-MSCs and aged-MSCs. **(B)** SA-β-Gal staining showed that senescence increased significantly in aged MSCs. **(C)** Representative immunoblot images and quantitative analysis of p21, p53, and p16 protein level in young and aged-MSCs. (*n* = 3). **(D)** Quantitation of cell cycle phases by propidium iodide staining. (*n* = 3). **(E)** The CCK-8 assay showed that aged MSCs grew more slowly than young MSCs. (*n* = 6). **(F)** Young and aged exosomes were observed using TEM. **(G)** The exosome surface markers were analyzed by Western blot. **(H)** Nanoparticle tracking analysis was used to analyze the particle size and concentration of Young-Exo and Aged-Exo. *p < 0.05; **p < 0.01; ***p < 0.001; ****p < 0.0001; NS, not significant.

Both young and aged MSCs were positive for MSC surface markers such as CD105, CD44, CD73, and CD90; and negative for CD45 and CD31 (Figure 1A). SA-β-Gal staining showed that senescence greatly increased in aged MSCs (Figure 1B and Supplementary Figure S1). Expression of senescent biomarker P21 and P53 significantly increased in aged MSCs compared with young MSCs but there was no significant difference in P16 protein expression (Figure 1C). The percentage of cells in G0 and G1 phase also increased significantly more in aged MSCs than young MSCs (Figure 1D). The aged MSCs grew more slowly than young MSCs at 3, 5, and 7 days after culturing with CCK-8 (Figure 1E). The characteristics of Exos derived from young and aged MSCs were detected by TEM, NTA and Western blotting. The diameter of most particles was around 110 nm and expressing surface markers included TSG101, CD81, and CD63 (Figures 1F–H). These results suggested that exosomes had been collected and purified successfully.

### Pro-angiogenesis and Anti-apoptotic Effects of Young-Exo *in vitro*

**FIGURE 2:**
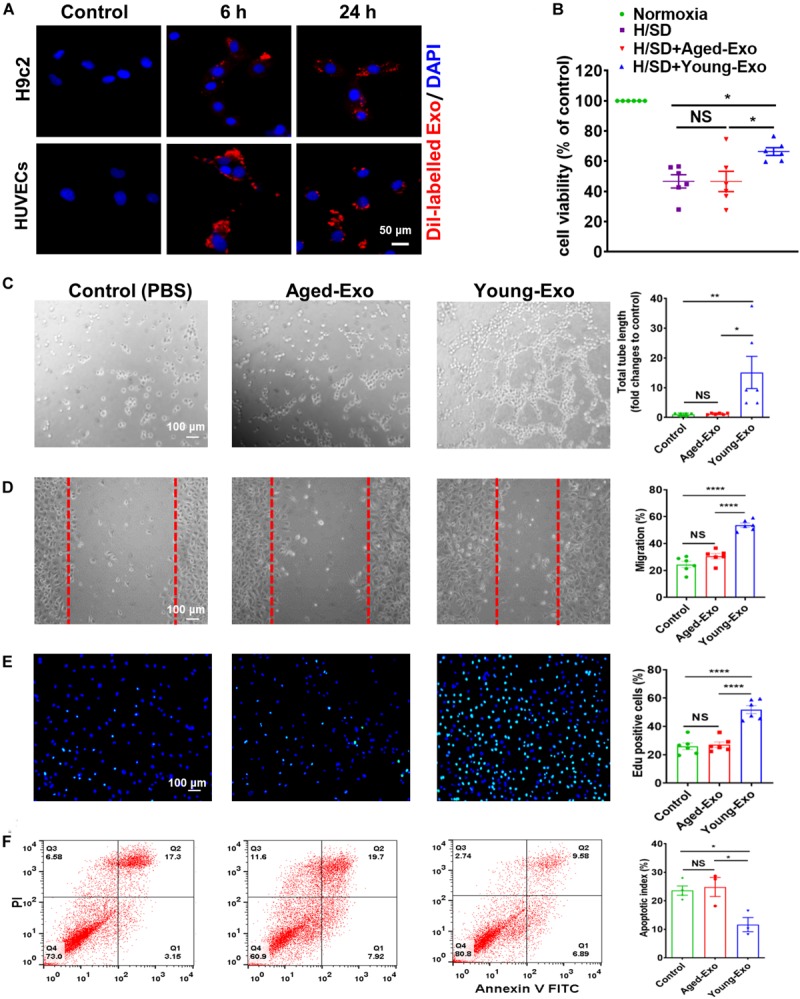
Internalization of exosomes in H9c2 and HUVECs cells, and effects of Young-Exo and Aged-Exo on recipient cells. **(A)** Dil-labeled exosomes (red) internalized by H9c2 and HUVECs was observed by confocal microscopy. The labeled exosomes were observed in the perinuclear region of recipient cells. **(B)** Cell viability of H9c2 cells was detected among four groups. (*n* = 6) **(C)** The tube formation ability of HUVECs was measured in the Young-Exo group, Aged-Exo group and control group (PBS) (*n* = 6). **(D)** The migration ability of HUVECs was tested among three groups (*n* = 6). **(E)** Proliferation rate was determined by Edu staining (DAPI, blue; Edu positive cells, green). (*n* = 6). **(F)** Apoptotic rate was determined by Annexin V-PI staining via flow cytometry among three groups. (*n* = 3). *p < 0.05; **p < 0.01; ****p < 0.0001; NS, not significant.

To further determine the roles of Exos derived from young and aged MSCs *in vitro*, Dil-labeled Exos were used to assess the internalization ability of Exos. After staining, washing and centrifuging, MSC-derived Exo were obtained and cultured with H9c2 cells and HUVECs. Red fluorescence was observed in the cytoplasm of cells at 6 and 24 h after treating with Dil-labeled Exos, while no red fluorescence was observed in the control group. This indicated that Exos could be taken up by cells and located in the cytoplasm (Figure 2A).

To evaluate the effects of Exos derived from young and aged MSCs (Young-Exo and Aged-Exo) *in vitro*, H9c2 cells and HUVECs were incubated with PBS (Control group), Aged-Exo and Young-Exo. Cell viability of H9c2 cells under H/SD significantly increased in the Young-Exo group compared with Aged-Exo and control groups (Figure 2B). In addition, apoptosis reduced significantly in the Young-Exo group compared with Aged-Exo and control groups (Supplementary Figure S2). The angiogenic tube formation ability in HUVECs was significantly enhanced in the Young-Exo group compared with Aged-Exo and control groups (Figure 2C) as was the migration ability of HUVECs. Nonetheless there was no significant difference of migration ability between the Aged-Exo group and control group (Figure 2D). Edu assay showed a significantly higher percentage of Edu-positive (proliferating) cells in the Young-Exo group than control and Aged-Exo groups (Figure 2E). We also found a significant reduction in apoptosis of HUVECs under H/SD condition in the Young-Exo group compared with Aged-Exo and control groups (Figure 2F). These results indicated that Young-Exo cells exhibited pro-angiogenesis, proliferation and anti-apoptotic effects *in vitro*, not evident for Aged-Exo cells.

### Young-Exo Cells Were Incapable of Effectively Maintaining Cardiac Function in Rats Post MI

**FIGURE 3:**
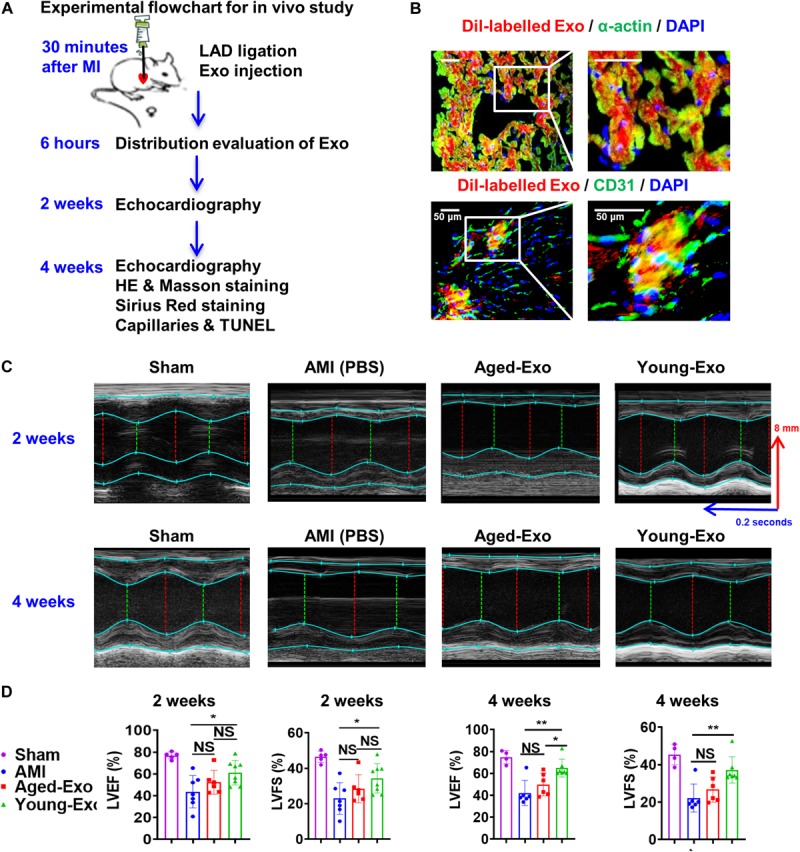
Cardiac function in a rat model of MI was preserved effectively by Young-Exo. **(A)** Schematic of *in vivo* experiment. **(B)** Distribution of Dil-labeled exosomes in the infarcted rat hearts. **(C)** Echocardiography analysis was performed at 2 and 4 weeks after establishment of MI. **(D)** LVEF and LVFS were enhanced significantly in Young-Exo group compared with Aged-Exo and AMI groups (2 weeks post MI: *n* = 5–8 for each group; 4 weeks post MI: *n* = 4–7 for each group). *p < 0.05; **p < 0.01; NS, not significant.

**FIGURE 4:**
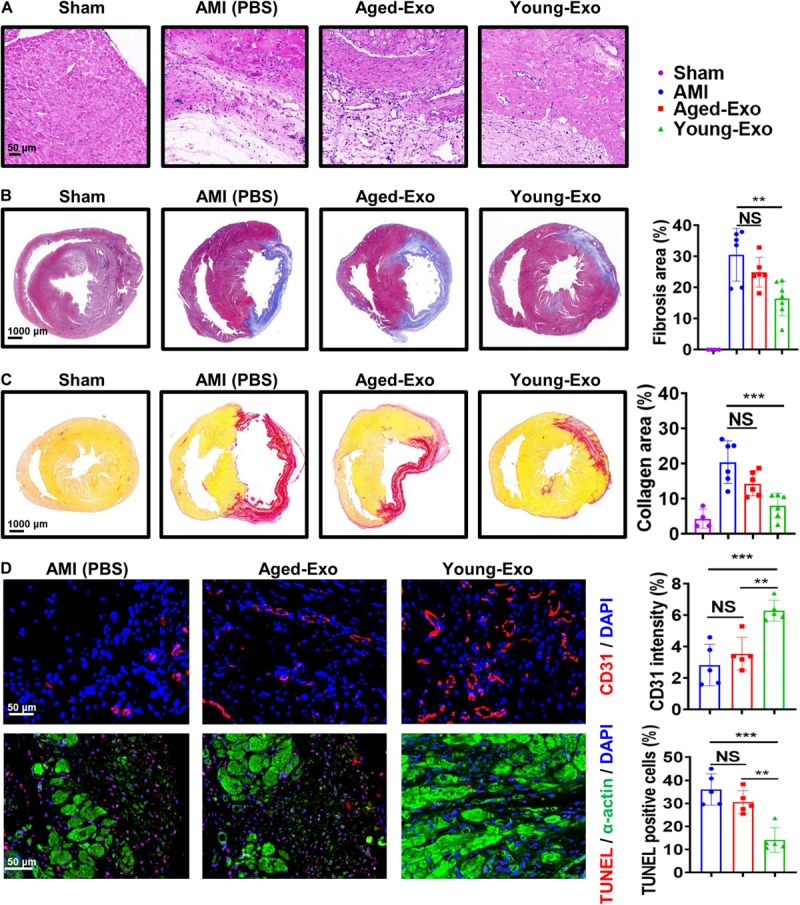
Aged-Exo were unable to inhibiting apoptosis or promote angiogenesis *in vivo*. **(A)** HE staining images at the border zone 4 weeks after MI. **(B)** Masson staining images at 4 weeks after MI. Red, myocardium; blue, scarred fibrosis. (*n* = 4–7 for each group). **(C)** Sirius Red staining images 4 weeks post MI among in the four groups (*n* = 4–6 for each group). **(D)** Representative fluorescence images of blood vessels in the border zone of ischemic hearts stained with CD31 (red). TUNEL showed less myocardium apoptosis in the Young-Exo group compared with Aged-Exo and AMI groups. (*n* = 5 for each group). **p < 0.01; ***p < 0.001; NS, not significant.

To assess the benefits of Exos *in vivo*, PBS (AMI group), Young-Exo, and Aged-Exo cells were injected into the infarction border zone 30 min after establishing a model of MI (Figure 3A). The distributions of Dil-labeled-Exos in the infarcted heart were observed 6 h post MI. Dil-labeled-Exos were detected in cardiomyocytes and endothelial cells (Figure 3B). LVEF and LVFS were slightly enhanced in Young-Exo group compared with the AMI group but not the Aged-Exo group. Four weeks post MI, LVEF, and LVFS significantly increased in the Young-Exo group compared with the AMI and Aged-Exo groups (Figures 3C,D). Inflammatory cell infiltration also significantly decreased in the Young-Exo group compared with the Aged-Exo and AMI groups (Figure 4A). Masson staining showed that the fibrotic area was significantly reduced in the Young-Exo group (Figure 4B). The collagen area was also significantly and consistently more reduced in the Young-Exo group than AMI group (Figure 4C). In addition, the capillary density dramatically increased compared with the Aged-Exo and MI groups. TUNEL also showed less myocardial apoptosis in the Young-Exo group compared with Aged-Exo group (Figure 4D). These results suggest that Young-Exo cells significantly enhanced the functional recovery of infarcted hearts, while Aged-Exo showed little beneficial effects.

### Exosomal miR-221-3p Level Decreased More in Aged-Exo Than Young-Exo

**FIGURE 5:**
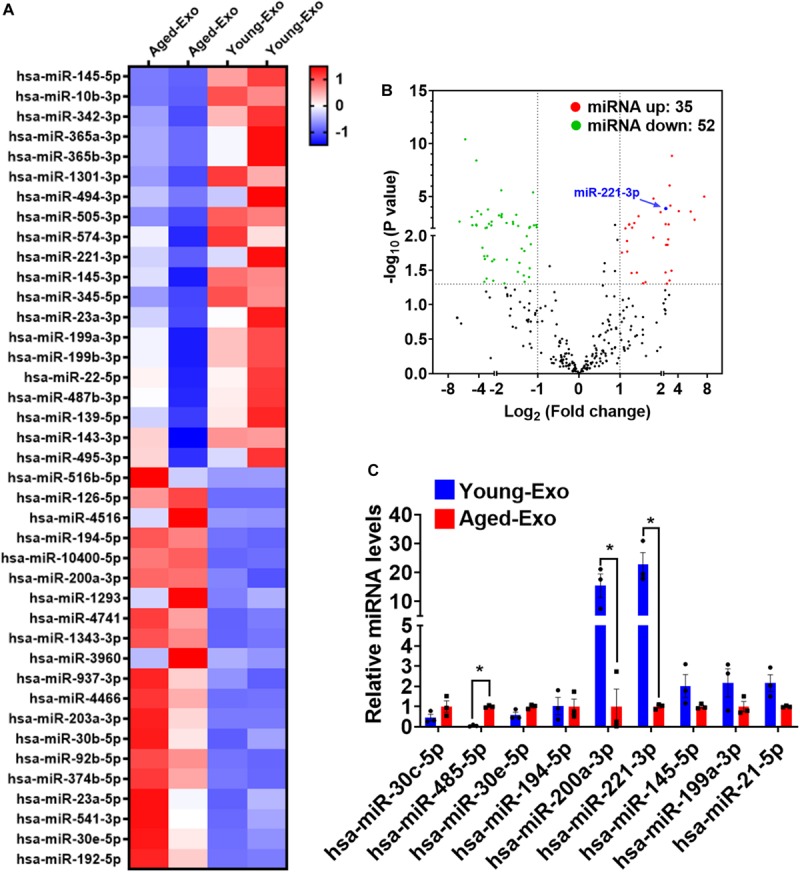
Exosomal miRNA expression profile of Young-Exo and Aged-Exo. **(A)** Heat map based on top 20 up-regulated and down-regulated miRNA expression (red: up-regulation, blue: down-regulation). **(B)** Volcano plot showing log_2_ (Fold change) (Young-Exo vs. Aged-Exo) on the x-axis and -log_10_ (P value) on the y-axis. miR-221-3p (blue) was significantly decreased in Aged-Exo. **(C)** Real-time PCR of some up-regulated and down-regulated miRNAs. (*n* = 3). *p < 0.05.

Increasing evidence suggests that miRNAs carried by Exos play a very important role in regulating cellular functions of recipient cells. To investigate the mechanism of Young-Exo-induced protective effects, we performed miRNA sequencing on exosomes derived from young and aged MSCs (Figure 5A). In total, 35 miRNAs were identified to be up-regulated in Young-Exo compared with Aged-Exo, while 52 miRNAs were identified to be down-regulated (Figure 5B). Real time PCR analysis confirmed four up-regulated miRNAs and five down-regulated miRNAs. In particular, miR-221-3p decreased significantly in Aged-Exo (Figure 5C). These data suggest that exosomal miR-221-3p may play a very important role during transition of Young MSCs to Aged MSCs.

### Exosomal miR-221-3p Played an Important Role in Angiogenesis and Cardiac Regeneration in Young-Exo

**FIGURE 6:**
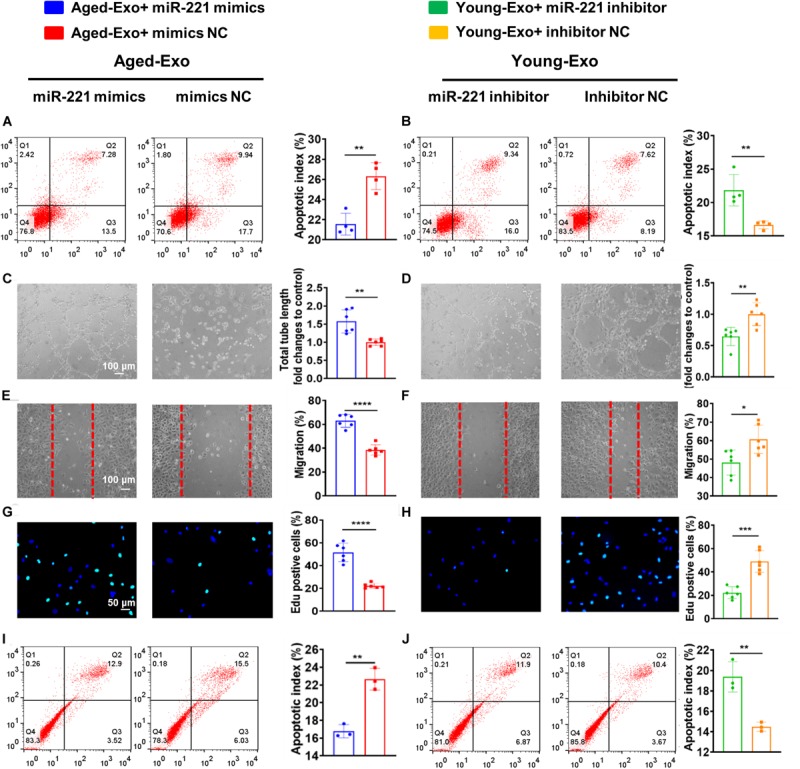
Exosomal miR-221-3p plays a very important role in Young-Exo-induced angiogenesis and cardiac repair. **(A)** Apoptotic rate was determined by flow cytometry in Aged-Exo treated H9c2 cells between miR-221-3p mimics group and mimics negative control group. (*n* = 4). **(B)** Apoptotic rate was determined by flow cytometry in Young-Exo treated H9c2 cells between miR-221-3p inhibitor group and inhibitor negative control group. (*n* = 4). **(C)** The tube formation ability of Aged-Exo-treated HUVECs between two groups. (*n* = 6). **(D)** The tube formation ability of Young-Exo-treated HUVECs between two groups. (*n* = 6). **(E)** The migration ability of Aged-Exo-treated HUVECs. **(F)** The migration ability of Young-Exo-treated HUVECs. **(G)** Edu assay were performed in Aged-Exo-treated HUVECs. **(H)** Edu assay was performed in Young-Exo treated HUVECs. **(I,J)** Apoptotic rate among different groups in HUVECs. (*n* = 3). **(C–H)** *n* = 6. *p < 0.05; **p < 0.01; ***p < 0.001; ****p < 0.0001.

In order to confirm that exosomal miR-221-3p plays a role in the beneficial effect of angiogenesis and cardiac regeneration in Young-Exo, miR-221-3p mimics and miR-221-3p inhibitor were successfully transfected into H9c2 cells and HUVECs, respectively (Supplementary Figure S3). Transfection of miR-221-3p mimics could enhance the anti-apoptotic effects of Aged-Exo cultured with H9c2 cells (Figure 6A). Knock down of miR-221-3p could attenuate the anti-apoptotic effects of Young-Exo incubated with H9c2 cells (Figure 6B). Additionally, miR-221-3p mimics could enhance angiogenesis, migration, and proliferation and also suppress apoptosis in Aged-Exo treated HUVECs (Figures 6C,E,G,I). In contrast, the anti-apoptotic and angiogenic effects of Young-Exo-treated HUVECs could be attenuated by transfection of miR-221-3p inhibitor (Figures 6D,F,H,J).

**FIGURE 7:**
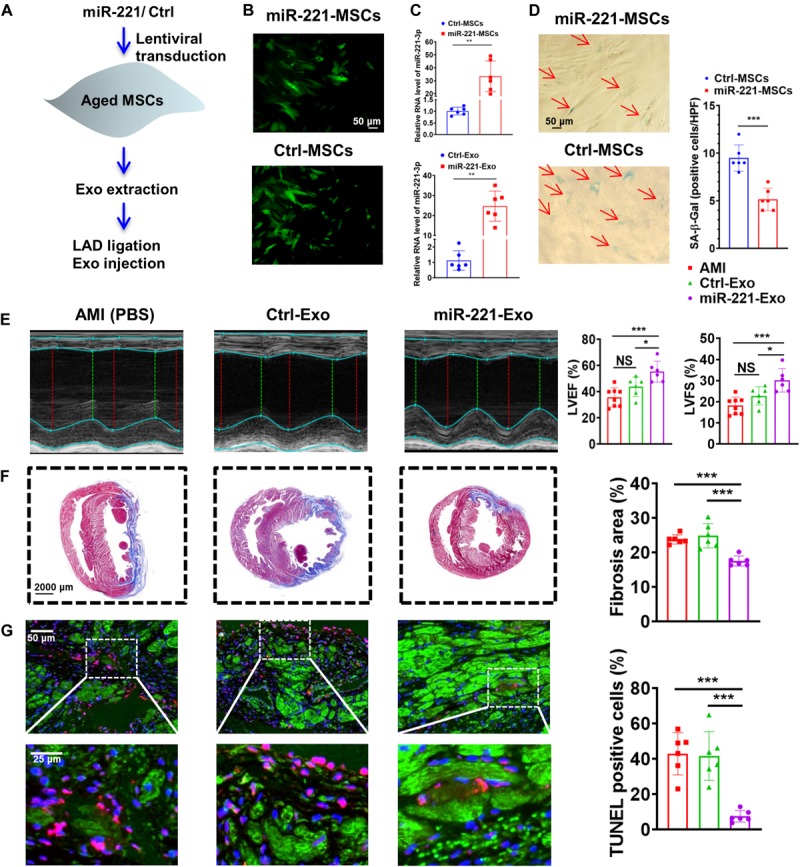
The effect of miR-221-3p loaded exosomes in the rat after MI. **(A)** The flowchart of lentiviral transduction and *in vivo* experiments. **(B)** Successful lentiviral transduction was confirmed by positive fluorescence signal under microscope. **(C)** Cellular and Exosomal miR-221-3p expression was validated by real-time PCR. (*n* = 6). **(D)** There were fewer SA-β-Gal positive cells per high power field (HPF) under microscopy (×200) in the miR-221-MSCs group. (*n* = 6). **(E)** Echocardiography analysis was performed 4 weeks post MI, and representative images are shown. LVEF and LVFS were significantly increased in the miR-221-Exo group compared with other groups. (*n* = 6–8 for each group). **(F)** Masson staining 4 weeks after MI. Red, myocardium; blue, scarred fibrosis. (*n* = 6). **(G)** TUNEL showed less myocardium apoptosis in the miR-221-Exo group compared with Ctl-Exo and AMI groups. (*n* = 6). *p < 0.05; **p < 0.01; ***p < 0.001; NS, not significant.

Aged MSCs were then infected with lentiviruses containing miR-221 and its negative control (Figure 7A). Green fluorescence was observed in both miR-221-MSCs (MSCs transfected with lentiviruses containing miR-221) and Ctrl-MSCs (MSCs transfected with lentiviruses containing miR-221 negative control) (Figure 7B). The cellular and exosomal expression of miR-221-3p significantly increased in the miR-221-MSC group compared with the Ctrl-MSC group (Figure 7C). There were fewer SA-β-Gal positive cells in the miR-221-MSC group than Ctrl-MSC group, indicating that senescence of MSCs could be alleviated by miR-221 overexpression (Figure 7D). We then extracted exosomes from miR-MSC and Ctrl-MSC groups. PBS, Ctrl-Exo (Exo derived from Ctrl-MSCs) and miR-221-Exo (Exo derived from miR-221-MSCs) were injected into the myocardium at the border area 30 min post MI. LVEF and LVFS significantly increased and fibrosis reduced 28 days post MI in the miR-221-Exo group compared with the Ctrl-Exo group (Figures 7E,F). In addition, TUNEL staining showed less myocardium apoptosis in the miR-221-Exo group compared with Ctrl-Exo and AMI groups (Figure 7G). These results indicate that miR-221-3p plays an important role in Young-Exo-induced angiogenesis and cardiac repair.

### miR-221-3p Decreased Pro-apoptotic and Increased Pro-angiogenic Protein Expression

**FIGURE 8:**
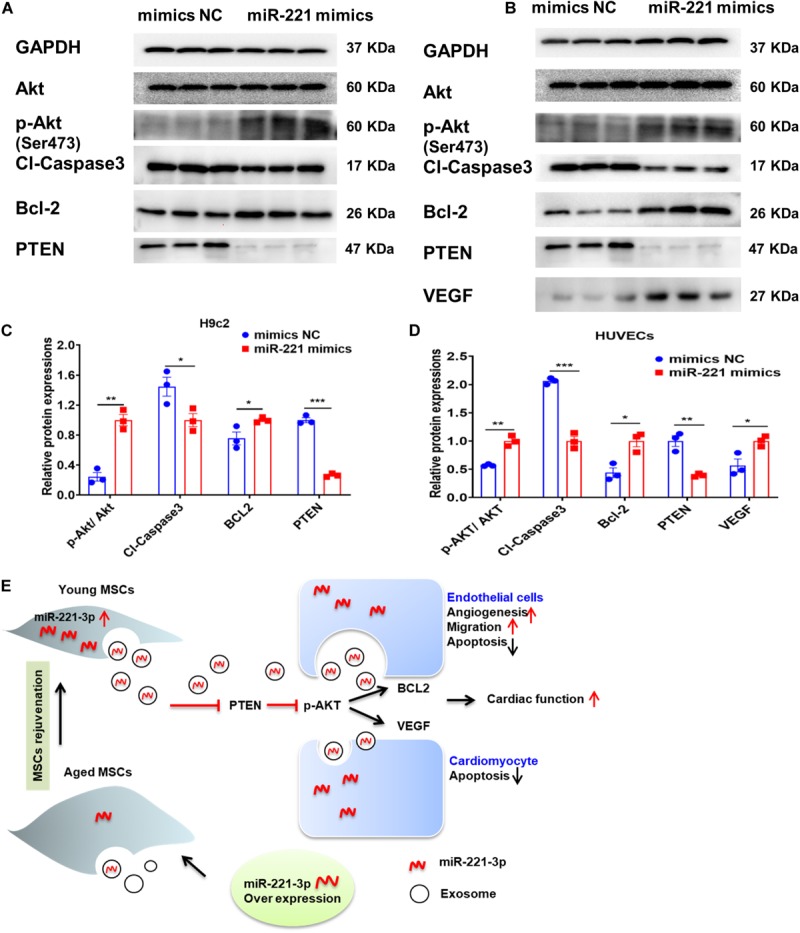
Exosomal miR-221-3p targets the PTEN/Akt signaling pathway after myocardial injury. **(A,B)** Western blot images showed the expression of proteins associated with the PTEN/Akt pathway in H9c2 **(A)** cells and HUVECs **(B)**. **(C)** Quantitative analysis of the levels of Akt, p-Akt, Cleaved caspase-3, Bcl-2, PTEN in H9c2 cells. (*n* = 3). **(D)** Quantitative analysis of the levels of Akt, p-Akt, Cleaved caspase-3, Bcl-2, PTEN, VEGF in HUVECs. (*n* = 3). **(E)** A proposed working model of this study. All miR-221 mimic groups were normalized to the relevant mimic negative control group. *p < 0.05; **p < 0.01; ***p < 0.001.

miR-221-3p was overexpressed with miR-221 mimics in H9c2 cells and HUVECs. We first observed enhanced Akt phosphorylation (Ser473) with up-regulation of miR-221-3p (Figures 8A–D). Next, proapoptotic protein, Cl-caspase-3 was down-regulated and anti-apoptotic protein Bcl-2 was up-regulated in the miR-221 mimics group in both H9c2 cells and HUVECs. The expression of reported target gene, PTEN was also decreased in the miR-221-3p mimics group compared with its negative control in both two cell lines. Additionally, protein expression of VEGF was increased in HUVECs. Taken together, our data indicated that miR-221-3p inhibited cardiomyocyte apoptosis and promoted angiogenesis by PTEN/Akt signaling.

## Discussion

This study revealed that Aged-Exo could not inhibit apoptosis, reduce fibrosis or promote angiogenesis and had a very weak effect on improving cardiac injury. The impairment of regenerative activities of Aged-Exo is at least partly due to down-regulation of miR-221-3p. Up-regulation of miR-221-3p in aged MSCs may be beneficial for improving their angiogenic, migration, proliferation abilities and in suppressing apoptosis through the PTEN/Akt pathway (Figure 8E).

Over the past decade, cell therapy has become a novel strategy for cardiac repair (Deuse et al., 2009; Zhang et al., 2011). Exosomes are important paracrine components, repairing tissue by encasing and sending RNAs and proteins (Phan et al., 2018; Shi et al., 2018). Previous study showed that exosomes from cardiac stem cells and mouse embryonic stem cells could afford the same cardioprotective effects as stem cells (Todorova et al., 2017; Liu et al., 2020). Nonetheless exosomes derive mainly from young donors and the contents of Aged-Exo cells may change and further reduce their reparative ability. In this study, Aged-Exo cells lost their proangiogenic drive of HUVECs tube formation and failed to inhibit cardiomyocyte apoptosis in injured rats’ hearts. This evidence suggests a dysfunction of Aged-Exo in ischemic tissue repair. Our findings may explain the compromised therapeutic effects in clinical trials that use autologous stem cells (Mathiasen et al., 2015, 2019). Aged MSCs have altered exosomal content that renders stem cells unable to affect therapeutic repair. Thus rejuvenation of aged MSCs in the elderly (Boulanger et al., 2017) population is of great interest.

The transfer of miRNAs and proteins from specific donor cells to recipient cells is a key mechanism for exosome-mediated repair (Boulanger et al., 2017; Todorova et al., 2017). In this study, the sequence examination of exosomal miRNAs showed that miR-221-3p was much higher in Exos secreted from young MSCs than aged MSCs. When miR-221-3p was delivered to cardiomyocytes, it could reduce apoptosis and enhance angiogenesis and cardiac function in a rat model of MI. After down-regulation of miR-221-3p in Young-Exo, these benefits were attenuated while up-regulation of miR-221-3p in Aged-Exo restored their therapeutic efficacy. Coskunpinar et al. reported that circulating miR-221 has a high discriminative value and significant relation with troponin and left ventricular systolic function. It may serve as a biomarker for early prediction of acute MI (Coskunpinar et al., 2016). Another study showed that circulating miR-221 is consistent with myocardial fibrosis and hypertrophy (Huang et al., 2020). MiR-221 also significantly reduced cardiomyocte H/R injury in association with inhibition of autophagy (Chen et al., 2016). Verjans et al. reported that the miRNA-221 level in patients with aortic stenosis correlated negatively with the extent of myocardial fibrosis and with left ventricular stiffness (Verjans et al., 2018). Wu reported that cancer cell-derived exosomal miR-221-3p promotes angiogenesis (Wu et al., 2019). These data support the important role of miR-221-3p in exosome-induced cardiac repair.

There are potential clinical implications for these findings. Unlike cell based therapeutic products, Exos provide a readily available and universal treatment option (Armstrong et al., 2017). No complicated steps are required for cryopreservation of Exos. The host immune system has good tolerance to the injection of allogeneic or even heterologous Exos (Jung et al., 2017). This study proves that compared with Young-Exo, Aged-Exo lack the ability of cardiac repair. Our findings may explain why autologous cell therapy in patients has shown limited therapeutic effects in some clinical trials (Fisher et al., 2015; Nguyen et al., 2016). In addition, the data of this study suggest that the dysfunction of Aged-Exo can be partly rescued by regulating specific microRNA in Exos. This study provides insight into the therapeutic potential of Exos and strategies to treat patients with defective exosomes by modulating specific microRNA.

This study has some limitations. First, HUVECs are not a good representation compared with primary coronary endothelial cells. Second, we found that protein levels of the target gene (PTEN) were altered by up-regulation of miR-221-3p. We did not conduct dual luciferase reporter assays. Nonetheless the direct combination of miR-221-3p and PTEN has already been confirmed in other studies (Li et al., 2016; Gong et al., 2019; Han et al., 2019). Third, the rats used in this study were relatively young. Their endogenous reparative ability may differ to that of elderly rats with long-term ischemic heart disease. Finally, although this study focused on miR-221-3p, there are several other miRNAs that are down-regulated in Aged-Exo and Young-Exo, such as miR-30c-5p, miR-485-5p and miR-200a-3p. These miRNAs may also contribute to the Young-Exo-induced cardiac protective effects post MI. Further studies are needed to clarify the function of other molecules in exosomes.

## Conclusion

The impaired regenerative abilities of Aged-Exo are due to down-regulation of miR-221-3p. Up-regulation of miR-221-3p in aged MSCs could improve their ability of angiogenesis, migration, proliferation and suppression of apoptosis through the PTEN/Akt pathway.

## Data Availability Statement

The raw data supporting the conclusions of this article will be made available by the authors, without undue reservation, to any qualified researcher.

## Ethics Statement

This study was approved by the ethics committee of The First Affiliated Hospital of Nanjing Medical University. All subjects gave written informed consent in accordance with the Declaration of Helsinki. All animal experiments were approved by the Institutional Animal Care and Usage Committee of Nanjing Medical University (No. IACUC-1905024).

## Author Contributions

LS, WZhu, and FZ contributed to the design of the study. LS, WZhu, PZ, JZ, YLi, and WZha performed the experiments. LS, WZhu, YLu, YZ, QC, and FZ contributed to writing the manuscript. LS and WZhu contributed to the material support of the study. All authors read and approved the final manuscript.

## Conflict of Interest

The authors declare that the research was conducted in the absence of any commercial or financial relationships that could be construed as a potential conflict of interest.
