# Supplementary material for: Down-Regulated Exosomal MicroRNA-221 – 3p Derived From Senescent Mesenchymal Stem Cells Impairs Heart Repair
Source: Front Cell Dev Biol. 2020 May 5;8:263. doi: 10.3389/fcell.2020.00263 (PMC7214920; doi:10.3389/fcell.2020.00263)
Supplement: Supplementary file 1 [file Table_1.pdf]

Supplementary table 1. Primer list

Primers	Sequences (5'–3')
hsa-miR-200a-3p Forward	AGCGTAACACTGTCTGGTAA
hsa-miR-200a-3p Reverse	TCCTCCTCTCCTTCCTTCTC
hsa-miR-194-5p Forward	GGGTGTAACAGCAACTCCA
hsa-miR-194-5p Reverse	TCCTCCTCTCCTTCCTTCTC
hsa-miR-30e-5p Forward	AGGGGTGTAAACATCCTTGAC
hsa-miR-30e-5p Reverse	TCCTCCTCTCCTTCCTTCTC
hsa-miR-145-5p Forward	GTGTCCAGTTTTCCCAGGA
hsa-miR-145-5p Reverse	GCGTTGTGTTGTGTTGTGTT
hsa-miR-485-5p Forward	AGAGGCTGGCCGTGAT
hsa-miR-485-5p Reverse	AGGGAGGAAGGAAGATAGGG
hsa-miR-30c-5p Forward	AGCGTGTAAACATCCTACACT
hsa-miR-30c-5p Reverse	TCCTCCTCTCCTTCCTTCTC
hsa-miR-221-3p Forward	GGGAGCTACATTGTCTGCTG
hsa-miR-221-3p Reverse	GAGAGGAGAGGAAGAGGGAA
hsa-miR-199a-3p Forward	GGGACAGTAGTCTGCACAT
hsa-miR-199a-3p Reverse	GAGAGGAGAGGAAGAGGGAA
hsa-miR-21-5p Forward	AGGGGTAGCTTATCAGACTGA
hsa-miR-21-5p Reverse	GTTGTGGTTGGTTGGTTTGT
U6 Forward	CTCGCTTCGGCAGCACA
U6 Reverse	AACGCTTCACGAATTTGCGT
Cel-miR-39-3p Forward	GGGTCACCGGGTGTAAATC
Cel-miR-39-3p Reverse	GAGAGGAGAGGAAGAGGGAA
