# Supplementary material for: Down-Regulated Exosomal MicroRNA-221 – 3p Derived From Senescent Mesenchymal Stem Cells Impairs Heart Repair
Source: Front Cell Dev Biol. 2020 May 5;8:263. doi: 10.3389/fcell.2020.00263 (PMC7214920; doi:10.3389/fcell.2020.00263)
Supplement: Supplementary file 2 [file Data_Sheet_1.docx]

**Supplementary Figure legends**

**Supplementary Figure 1. Increased SA-β-Gal positive cells were oberserved in Aged MSCs group.** A) Quantitative analysis of SA-β-Gal positive cells between young and aged MSCs (n=6). All data are mean ± SEM. Statistical analysis was performed with t test. *P < 0.05, **P < 0.01, NS, not significance.

**Supplementary Figure 2. Young-Exo reduced apoptosis rate in H9c2 cells under H/SD condition.** A) Apoptotic rate was determined by Annexin V-PI staining via flow cytometry among three groups. B) Quantitative analysis of the apoptotic rates was shown. (n=6). All data are mean ± SEM. Statistical analysis was performed with one-way ANOVA followed by Tukey’s test. *P < 0.05, **P < 0.01, NS, not significance.

**Supplementary Figure 3. miR-221-3p mimics or inhibitor transfected target cells sucessfully.** A-B) Real-time PCR showed that miR-221 mimics (A) or inhibitor (B) transfected HUVECs sucessfully. C-D) Real-time PCR showed that miR-221 mimics (C) or inhibitor (D) transfected H9c2 cells sucessfully. (n=3). All data are mean ± SEM. Statistical analysis was performed with t test. *P < 0.05, **P < 0.01.
