# Supplementary figures and images for: Down-Regulated Exosomal MicroRNA-221 – 3p Derived From Senescent Mesenchymal Stem Cells Impairs Heart Repair
Source: Front Cell Dev Biol. 2020 May 5;8:263. doi: 10.3389/fcell.2020.00263 (PMC7214920; doi:10.3389/fcell.2020.00263)

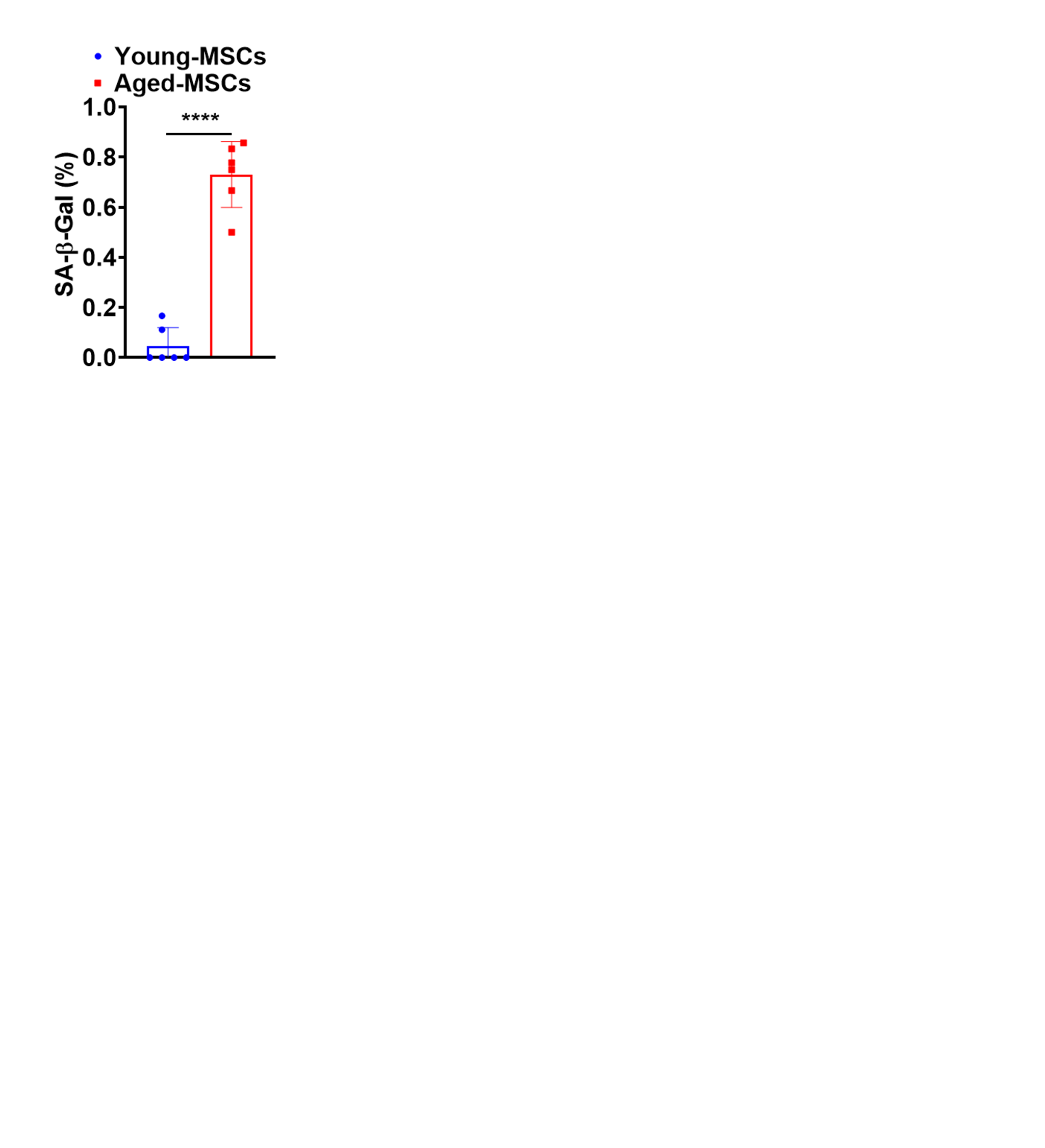

Supplement: Supplementary file 3 [file Image_1.tif]

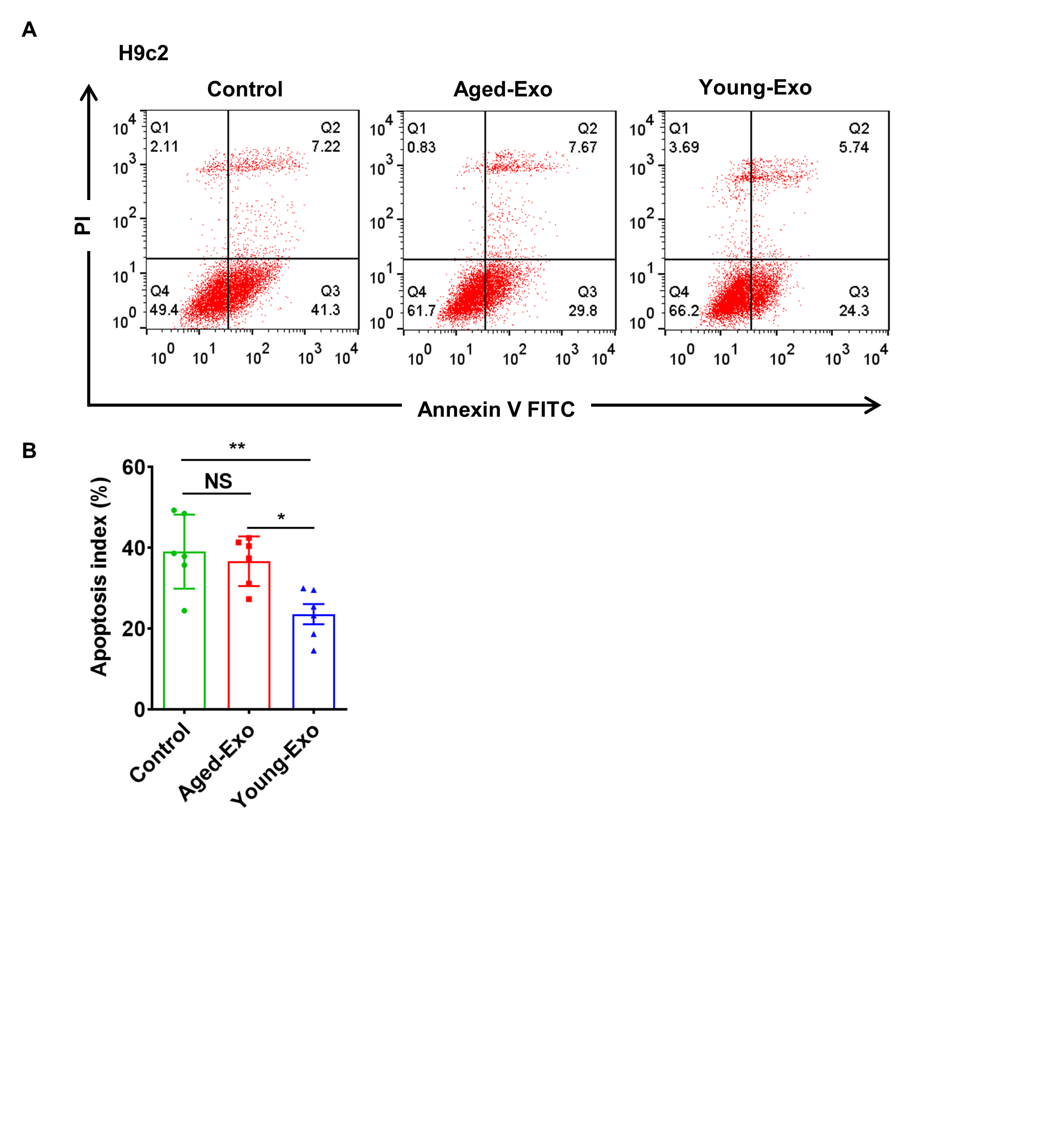

Supplement: Supplementary file 4 [file Image_2.tif]

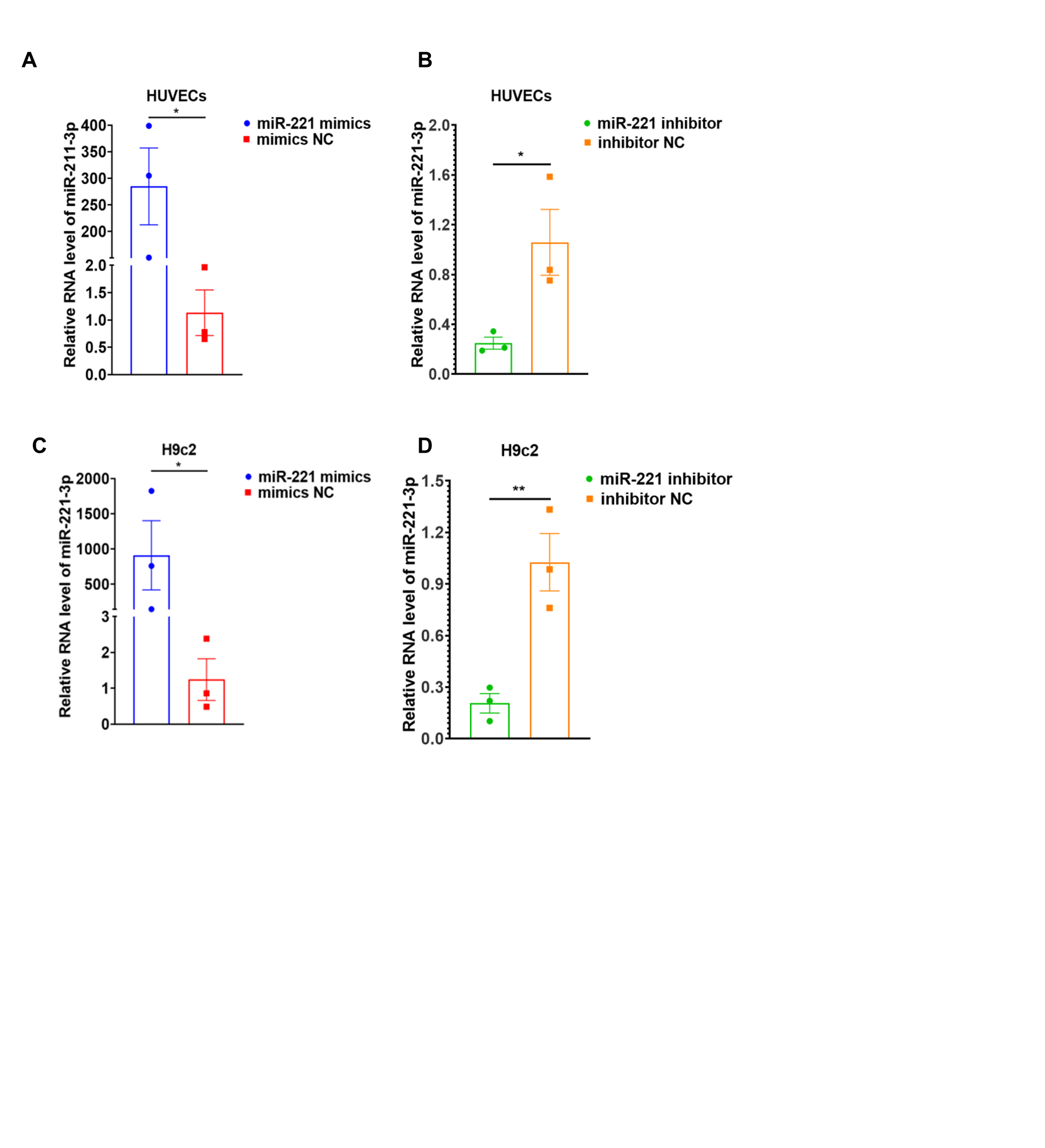

Supplement: Supplementary file 5 [file Image_3.tif]
